# The Examination of Blood Smear as a Diagnostic Asset

**Published:** 1916-04

**Authors:** Martha A. Wood

**Affiliations:** Houston, Texas


### The Examination of the Blood Smear as a Diagnostic Asset.

MARTHA A. WOOD, M. D., HOUSTON, TEXAS.

For the past ten years or more an examination of the blood smear has been more or less a routine with us. Looking backward over our experiences has but confirmed my opinion of its value as a diagnostic "sign post." The ease with which a smear may be made, and examined personally, or transferred to a pathologist, makes one wonder why recourse to such a procedure is not more in vogue.

The elements of a blood smear need but a brief mention. Of the red cells, we note their variations in size, shape, color, nucleation, etc. Of the blood plaques, we may get an approximate idea of their number.

The parasites of the blood may be seen, such a malaria, filaria, spirillae of relapsing fever, etc.

Of the leucocytes, we note the kinds (normal and abnormal) and the proportion of each, and we may estimate fairly accurately the total number per cubic mm.

The examination of the blood smear may establish a definite diagnosis of the disease, or it may be merely a symptom or "sign post" suggesting the way to a diagnosis.

The diseases in which a blood smear establishes a definite diagnosis (malaria, filiariasis, lymphatic and spleno-medullary, leukemia, etc.) are in the minority as regards the importance of a blood examination. Sometimes, however, decided errors in diagnosis are cleared up; as, for instance, cases of malarial fever simulating appendicitis. Two of such cases coming to our notice during the past fall showed malarial parasites and differential counts which precluded the possibility of any disease of an infectious nature, the counts being, respectively:

Case 1.—P., 70 per cent.; S. M, 18 per cent; L. M., 12 per cent; E., 0 per cent; B., 0 per cent; leucocytes scarce in smear. Total count, 4000.

Case 2.—P., 68 per cent; S. M., 22 per cent; L. M., 10 per cent; E., 0 per cent; B., 0 per cent; leucocytes scarce in smear. Total count, 6500.

Those diseases which influence the proportion of the different leucocytes in the blood, or, in other words, the differential leucocyte count, are the ones which in my opinion are most important for our consideration.

Typhoid fever, uncomplicated, at the beginning of the second week practically always shows a scarcity of leucocytes in the smear, a low percentage of polynuclears, an increase in the percentage of small mononuclears, and an absence of eosinophiles. To my mind, one gets much more diagnostic information at such a time from the differential count than from the Widal test, the latter so often proving negative until late in the case. Yet pathologists continue to get requests for a Widal test with an absolute neglect of the differential count. Every drop of dried blood for a Widal should be accompanied by a smear for a differential count to get the best help from the laboratory in such cases.

Complications in typhoid, such as pneumonia, perforation, etc., cause a corresponding change in the blood picture, making the differential resemble a septic count, and increasing the total number of leucocytes in the smear.

A case recently seen in the third week of typhoid, after a broncho-pnuemonia had developed showed: Polynuclears, 85 per cent; small mononuclears, 8 per cent; large mononuclears, 7 per cent; eosinophiles, 0; basophiles, 0. After the subsidence of the pneumonic symptoms the blood picture changed to: P., 65 per cent; S. M., 33 per cent; L. M., 2 per cent; E., 0; B., 0; a typical typhoid differential. In malaria, a large mononucleosis is usual.

Of diseases in which an eosinophilia presents itself, infection with intestinal parasites is the most important. Many times the discovery of an increase of eosinophiles in the blood smear has led to a successful examination of the feces for animal parasites. Exceptionally good results have been obtained by us in cases of taenia nana and of uncinaria infection with obscure nervous symptoms. A few hookworm cases have presented eosinophile counts of as high as 35 to 45 per cent. The total number of leucocytes in these cases is usually about normal or below normal.

In another group of cases, we find a leucocytosis more or less marked, an increase in polynuclears to between 75 to 85 per cent, but a normal or increased percentage of eosinophiles. Such a blood picture suggests a search for subacute gall bladder mischief or subacute appendicitis, or infection of the pelvis of the kidney, or gonococcic infection of the genital organs, or perhaps cancer of the intestines. It is, of course, necessary to exclude intestinal parasites as a complicating agency in causing the presence of eosinophiles, and thus masking a true "septic count."

Last of all, the most important to the general practitioner who has a surgical case to diagnose, is the so-called "septic count," i. e. an increase in the total number of leucocytes, an absence of eosinophiles, and a more or less marked increase in the percentage of polynuclear neutrophiles.

In this connection, one must remember that the offending toxins must have a free access to the general circulation to give the blood picture. Well walled-off abscesses, for instance, might give a distorted blood picture.

The percentage of polynuclears bears a direct ratio to the severity of the infection.

Looking over our cases of "septic counts," I find no flaws in the blood picture in any of our cases of a fulminating character. Cases of appendicitis where delay is dangerous always give warning in the typical septic count, and successive counts at intervals of three or four hours will show the progressive tendency of the case. This is especially true of the cases where delay means early abscess formation, or perforation and a peritonitis more or less general, with fatal results at times.

The most extreme case of this type in our series was one in which operation four hours after the attack began showed an appendix tense with pus. After removing the appendix and loosing the clamps on its base the pus shot out about three feet, as though from a squirt gun. This case showed a polynucleosis of 97 per cent, but the eosinophiles showed .5 per cent. I judge the short time between the beginning of the attack and the operative procedure did not permit of the total disappearance of the eosinophiles. Another case in a child 8 years old showed twelve hours after the first symptoms a polynucleosis of 98 per cent, no eosinophiles. Operation was refused, but was performed by another physician thirty-six hours after the onset, with fatal outcome from perforation and peritonitis. Unfortunately, our records show many cases of appendicitis where a "watchful waiting" policy inaugurated by the family physician has resulted in a long drawn out battle between the body forces and the toxins, with the toxins sometimes the winners in the battle, when early surgical intervention might have easily won the victory for the patient.

I am sure that the early blood pictures in these cases would have shown how much a surgeon's help was needed by the body forces.

In determining surgical conditions due to a less severe infectious agent, the blood picture is not as easy to interpret correctly. However, even in such cases, close correlation of the blood picture with the clinical symptoms rarely leads one astray.

In presenting these remarks, I have but touched the high places with regard to an interpretation of the findings of a blood smear. Any good text-book on diagnostic methods will complete these observations for you.

The material upon which these remarks have been based has come mainly from the practice of Dr. John T. Moore, of Houston, with whom I am associated.

---

Dr. Wilfred Grenfell, who is in charge of a division of the hospital unit that has been sent to France by Harvard University, has been termed "the doctor with the biggest practice in the world." His work as missionary among the fishermen in the northwest Atlantic has made him responsible for the physical as well as the spiritual welfare of some 30,000 men.

---

It has been announced that a fund has been established by Samuel Mather, of Cleveland, to found a school for the graduate study of tuberculosis. It is to be a memorial to the late Dr. E. L. Trudeau, who was the first to put to practical use the present rational method of treatment of tuberculosis. The school will probably be located at Saranac, N. Y., where Dr. Trudeau's sanitarium was situated.

